# An Analysis of Hip and Knee Joint Movement Characteristics in Overweight Individuals During Sit-to-Stand Transfers—Based on Statistical Parametric Mapping: An Exploratory Study

**DOI:** 10.3390/life16030515

**Published:** 2026-03-20

**Authors:** Guohui Zhao, Feifei Ma, Lei Li

**Affiliations:** 1 School of Physical Education, Shanxi University, Taiyuan 030006, China; zgh1990@sxu.edu.cn (G.Z.);; 2 School of Sport Science, Beijing Sport University, Beijing 100084, China

**Keywords:** overweight population, sit-to-stand transfer, motion analysis, statistical parametric mapping

## Abstract

Objective: The objective of this study was to explore the motion characteristics and movement strategies of the hip and knee joints in overweight individuals during sit-to-stand (STS) transfers using statistical parametric mapping (SPM). Methods: Twenty subjects were divided into an overweight group (*n* = 10) and a normal-weight group (*n* = 10) based on body mass index (BMI). The Qualisys infrared motion capture system and Kistler three-dimensional force platform were used for motion data collection, and Visual 3D and Matlab were used to calculate the angles and torque indicators of the lower limb hip and knee joints. Results: During the STS process, the maximum hip flexion angle of the overweight group was smaller than that of the control group (Z = −1.83, *p* = 0.043, r = 0.39), while the maximum abduction and external rotation angles were greater than those of the control group (Z = −2.15, *p* = 0.022, r = 0.46; Z = −2.02, *p* = 0.028, r = 0.48). SPM analysis showed that during the 0–52% phase of the hip joint in the frontal plane, the abduction amplitude of the overweight population was greater than that of the normal population (*p* < 0.05). The minimum external rotation angle of the knee joint was less than that of the control group (F(1,18) = 9.135, *p* = 0.043). The peak internal adduction and abduction torque of the hip joint in the overweight group was greater than that of the control group (Z = 2.37, *p* = 0.017, r = 0.54). Conclusions: Compared with the normal-weight population, the overweight population exhibited distinct motion characteristics of the hip and knee joints during the STS, with particularly pronounced differences in the hip joint. To maintain stability during STS, the overweight population adopts a compensatory movement strategy featuring a wider base of support via hip abduction and increased muscular torque to control frontal plane stability, which imposes greater functional loads on the hip joint. BMI-related movement characteristics should be studied in young adults under controlled experimental conditions, and future studies are needed to verify whether similar patterns exist in older adults.

## 1. Introduction

According to the World Health Organization, as of 2022, approximately 43% of adults worldwide are at risk of being overweight [1]. Overweight individuals not only have an increased risk of cardiovascular diseases but also have an increased risk of falls and a younger onset of lower limb mobility impairments, joint pain and osteoarthritis [2,3].

Research has shown that overweight individuals exhibit significant differences in body shape and athletic performance compared to those with normal weight [4,5,6]. The increase in muscle and fat in overweight individuals alters body morphology, causing the center of mass to shift forward, adversely affecting daily activities [7,8,9] such as walking and sit-to-stand (STS) movements [10]. Furthermore, the range of motion of overweight individuals may be limited due to postural characteristics, weakening their ability to control balance, thereby increasing the risk of falls [11,12]. Among various dynamic tasks, STS is a functional task performed at least 50 times a day by each person, effectively reflecting the lower limb functional status of different populations [13,14,15]. During STS, movement execution relies on the coordination between forward momentum generation and anti-gravity support. In the pre-seat-off phase, the hip joint primarily generates forward trunk momentum, allowing the COM to be transferred anteriorly over the base of support, thereby overcoming the mechanical constraint imposed by the seated posture and completing weight transfer. In the post-seat-off phase, the body is fully supported by the lower limbs, and the knee extensors become the main power source, producing extensor torque to provide vertical propulsion and body weight support and elevate the body to an upright position. Given these phase-dependent and complementary biomechanical functions, examining hip and knee joint mechanics is essential for understanding movement strategy adaptations during STS. Therefore, this study primarily explores the differences between the hip and knee joints.

Previous studies have often focused on extracting extreme values or other discrete data for a comparative analysis of continuous motion characteristics [16,17,18], without fully considering the biomechanical patterns of movement strategy changes across the entire time dimension. Statistical parametric mapping (SPM), as a testing method suitable for continuous data, addresses biases in traditional statistical tests of continuous data and has been widely applied in the statistical analysis of one-dimensional continuous variables in sports biomechanics [19,20]. Therefore, this study, based on SPM one-dimensional multivariate vector field analysis, combined with motion characteristic analysis during STS, aims to explore the changes in lower limb motion loads in overweight populations during STS, providing a theoretical basis for injury prevention in this population. We hypothesize that overweight individuals will exhibit greater frontal plane motion (hip abduction) and increased hip muscle torque to maintain stability during the STS transfer compared to normal-weight individuals.

## 2. Materials and Methods

### 2.1. Participants

According to the Chinese national criteria for adult weight status (WS/T 428-2013) [21], which was confirmed and recommended in the latest Chinese guidelines on weight management [22], BMI was calculated as body weight (kg) divided by height squared (m^2^). Twenty college students were recruited and divided into an overweight group (24–27.9 kg/m^2^) and a normal-weight group (18.5–23.9 kg/m^2^) based on body mass index (BMI), with 10 individuals in each group. All participants completed a standardized weekly physical activity questionnaire (IPAQ), and total physical activity was calculated as MET-min/week. An independent sample *t*-test showed no significant difference between the overweight group (4629.5 ± 3024.7 MET-min/week) and the normal-weight group (3214.4 ± 2139.7 MET-min/week) (t = 1.13, *p* = 0.28, Cohen’s d = 0.54). Basic information is shown in Table 1.

All subjects had no specialized sports training or athletic level; no sensory or neurological disorders; and no history of lower limb joint surgery, meniscus injury, or ligament injury and had not engaged in vigorous exercise within 72 h prior to testing, with no muscle fatigue. Informed consent was obtained from all subjects involved in this study.

### 2.2. Data Collection and Processing

This experiment was conducted at the Sports Rehabilitation Medicine Center of Beijing Sport University using an 8-camera infrared motion capture system (Qualisys, Gothenburg, Sweden, sampling frequency 200 Hz) and 4 three-dimensional force platforms (Kistler, Winterthur, Switzerland, sampling frequency 1000 Hz) for data collection.

#### 2.2.1. STS Testing Procedure

Before testing: A height-adjustable chair without armrests or backrests was placed on two force platforms, and the seat height was individually adjusted to 100% of each subject’s lower leg length. Initial posture adjustment: Subjects were instructed to place their hands on their abdomen, keep their torso vertical to the ground, and position their feet on the two force platforms, with the distance between them equal to the width of the anterior superior iliac spine, toes externally rotated 15°, and ankles dorsiflexed to 75°. The sitting depth was half the length of the thigh, aligned with the front edge of the seat. All participants familiarized themselves with the experimental procedure and performed practice trials prior to formal testing, ensuring consistent movement patterns and minimizing potential learning effects. Official testing: After maintaining a stable sitting posture for 5 s, subjects were verbally instructed by the tester to stand up, requiring them to rise at a self-selected speed in a comfortable and natural manner, without moving their feet during the standing process, and to maintain an upright position for 15 s after standing. The initial posture was adjusted before each STS trial to ensure standardized starting conditions. Each participant performed repeated trials until five valid trials were obtained. A trial was considered valid when the participant completed the STS movement continuously and naturally without foot repositioning, an interruption of movement, or the loss of balance. A total of 5 valid data collections were made. Specifically, the STS task was divided into two phases. The start of movement was defined as the instant when the velocity of the acromion marker exceeded 0.1 m/s, indicating the initiation of trunk motion. The seat-off event was identified when the vertical ground reaction force measured by the force plate under the chair decreased below 10 N. The post-seat-off phase extended from the seat-off instant to the end of movement, which was defined as the moment when the acromion marker velocity fell below 0.01 m/s, indicating full trunk extension and stabilization.

#### 2.2.2. Data Processing

Qualisys Track Manager 2023.3 was used to identify the spatial coordinates of the markers, and a fourth-order Butterworth low-pass filter was applied, with a cutoff frequency of 6 Hz. C3D data were exported, and a human model was established in Visual 3D 4.0 (C-Motion, Germantown, MD, USA) to calculate the angles and torque data of the lower limb hip and knee joints. Joint angles were defined as follows: the upright position of the hip and knee joints was defined as 0°, with hip and knee flexion, adduction, and internal rotation defined as positive values and extension, abduction, and external rotation defined as negative values. All data were normalized to a complete sit–stand movement of 101 data points using spline interpolation in Matlab R2024a (MathWorks, Natick, MA, USA).

### 2.3. Statistical Analysis

In this study, custom MATLAB code (MathWorks, USA) in combination with the open-source SPM1d software (version M.0.4.10) package (www.spm1d.org) was used to perform statistical parametric mapping analyses. The normality of waveform residuals was first assessed using the built-in function spm1d.stats.normality.ttest. Based on the normality results, either parametric SPM1d or nonparametric SnPM1d was selected. Independent sample one-dimensional SPM{t} tests were then conducted separately for each kinematic and kinetic time series of the hip and knee joints in each anatomical plane during the STS task to compare the overweight and normal-weight groups [23,24]. In this study, joint angle and torque maximum and minimum data were subjected to Shapiro–Wilk normality tests; normal data were analyzed using independent sample *t*-tests, while non-normal data were analyzed using Mann–Whitney U tests. Differences were considered statistically significant when *p* < 0.05 and highly significant when *p* < 0.01.

## 3. Results

### 3.1. Temporal Parameters Between the Overweight Group and the Normal-Weight Group

The overweight group showed a significantly shorter total STS completion time than the normal-weight group (t = −2.36, *p* = 0.030, Cohen’s d = 1.08). No significant difference was observed between groups in the pre-seat-off phase (*p* = 0.627, Cohen’s d = 0.23). However, in the post-seat-off phase, the overweight group also demonstrated a significantly shorter completion time compared with the normal-weight group (t = −3.43, *p* = 0.003, Cohen’s d = 1.60) (Table 2).

### 3.2. Differences in Joint Angle Changes Between Overweight and Normal-Weight Groups

During STS, the overweight group exhibited a smaller maximum hip flexion angle than the control group (Z = −1.83, *p* = 0.043, r = 0.39). In contrast, the overweight group showed greater maximum hip abduction and greater minimum external rotation angles (Z = −2.15, *p* = 0.022, r = 0.46; Z = −2.02, *p* = 0.028, r = 0.48).

**Figure 1:**
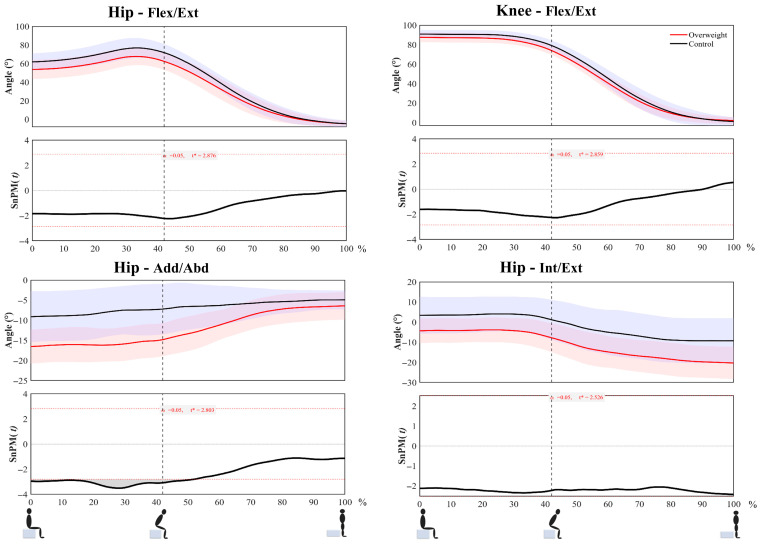
Joint angle time series and SPM test results during STS. (Red line represents overweight. Black line represents normal-weight group. t* denotes the critical threshold of the SPM{t} test at a significance level of α = 0.05).

SPM analysis further indicated that, between 0 and 52% of the STS cycle, hip abduction in the frontal plane was significantly larger in the overweight group than in the normal-weight group (*p* < 0.05) (Table 3, Figure 1).

### 3.3. Differences in Torque Changes Between Overweight and Normal-Weight Groups

**Figure 2:**
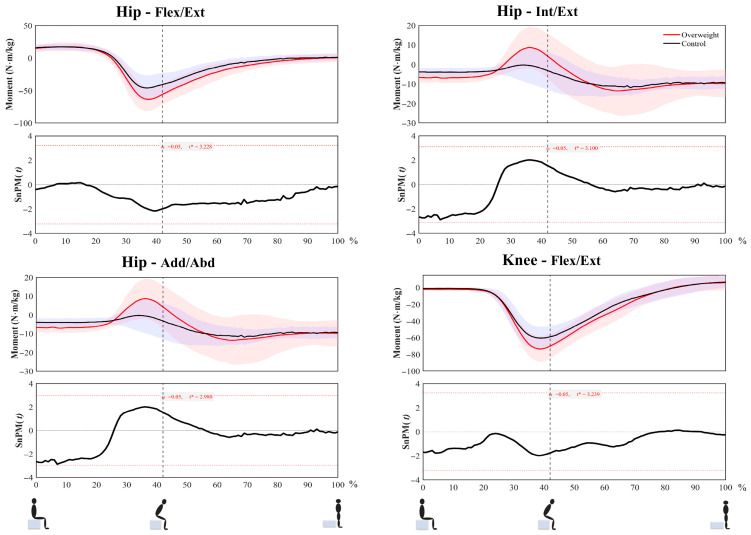
Torque time series and SPM test results during STS. (Red line represents overweight. Black line represents normal-weight group. t* denotes the critical threshold of the SnPM{t} test at a significance level of α = 0.05).

During the STS process, the peak internal adduction and abduction torque of the hip joint in the overweight group was greater than that of the control group (Z = 2.37, *p* = 0.017, r = 0.54), while no other results showed statistical differences (Table 4, Figure 2).

## 4. Discussion

In daily activities, STS is a movement that requires high lower limb load and is frequently used [25,26]. Analyzing single motion characteristics at specific moments may overlook changes in various aspects of the entire movement process and load distribution. Therefore, this study reveals the differences in joint angles and torques between overweight and normal populations during STS through a feature analysis of motion changes combined with SPM analysis, aiming to comprehensively analyze the unique biomechanical adaptations of overweight individuals when performing this action.

This study found that during STS, the maximum hip flexion angle of the overweight population was less than that of the normal-weight group. This may be related to the forward tilt of the pelvis in overweight individuals to maintain the body’s center of mass [27,28] and can also be attributed to the physical obstruction from excess abdominal mass, which directly limits the range of motion during deep hip flexion. Previous studies have shown that the position of the center of mass is crucial for dynamic balance regulation, and weight gain leads to changes in the center of mass position, requiring additional hip joint movement to maintain standing stability [29,30,31]. To unify the motion patterns of the subjects, this study limited the foot position and initial knee flexion angle of all subjects. In the same sitting posture, the overweight group exhibited a forward pelvic tilt to maintain sitting balance, resulting in a smaller maximum hip flexion angle compared to the normal-weight group.

Additionally, this study found significant differences in the starting positions of the STS movement between the overweight and normal-weight groups. The overweight group often exhibited hip joint abduction and external rotation postures, while the normal-weight group displayed abduction and internal rotation postures. Normal-weight individuals tend to adopt hip joint postures of abduction and internal rotation to distribute body weight and reduce pressure on the hip joint [12,32]. However, increased body weight exerts greater mechanical pressure on the joints and surrounding structures, leading overweight individuals to adopt additional movement patterns to alleviate the burden on lower limb joints [33,34]. Thus, the differences in hip torque between groups are likely the result of the combined effect of increased body mass and altered movement strategies, rather than being driven by a single factor. Huffman et al. [15] found that individuals with a BMI over 30 kg/m^2^ had a hip joint abduction angle that was 50% higher than that of the normal-weight group, and the abduction joint torque was approximately twice that of the normal-weight group. The SPM analysis results of this study further confirmed this, revealing that the overweight group significantly increased hip joint abduction during the 0–52% phase, with the maximum abduction angle being significantly higher than that of the normal-weight group. This indicates that overweight individuals may employ a greater abduction strategy to address balance issues in sitting posture. During hip joint abduction, hip abductor muscles contribute to enhancing frontal plane stability by widening the base of support, which may reduce compensatory knee joint loading and patellofemoral stress [35,36]. However, the peak internal adduction torque of the hip joint in the overweight group was significantly greater than that of the normal-weight group; after initial hip abduction widens the base of support, this greater torque reflects increased effort from the adductor muscles to control femoral motion and prevent excessive lateral sway as the center of mass rises during STS [37,38]. Therefore, the overweight population primarily compensates for the mechanical pressure on the joints caused by increased body weight through increased hip joint abduction and adductor muscle torque before standing up, a strategy that helps achieve stable forward thrust during standing but may also accelerate degenerative changes in the hip joint, leading to the occurrence of osteoarthritis [39,40].

From an anatomical perspective, the knee joint only exhibits in-plane motion in a flexed position [41], but in biomechanical testing, angle definitions are based on the coordinate axes between different segments, which may yield data on other anatomical planes. This study found that the maximum extension torque of the knee joint in the overweight group was less than that of the normal-weight group, showing a trend toward statistical significance (*p* = 0.065); the maximum flexion torque of the hip joint was also smaller than that of the normal-weight group, showing a trend toward statistical significance (*p* = 0.053). Sibella et al. [42] found that obese individuals tend to reduce hip joint torque while increasing knee joint torque during STS. However, this study found that the overweight group tended to reduce both the maximum torque of the hip joint and the maximum torque of the knee joint. This may be because although the body weight of overweight individuals is increased compared to normal individuals, their strength and control capabilities relative to obesity may still be sufficient to avoid the need for a significant increase in knee joint torque to support stability. Additionally, the increase in hip joint internal adduction torque may have compensated for the insufficient knee joint extension torque, which may also increase instability on the medial and lateral sides of the lower limbs [43]. This supports the view that individuals with a higher BMI have a greater tendency for lateral displacement when transitioning from static to dynamic postures, with weaker postural control abilities and a higher risk of falls.

Limitations: The conclusions primarily reflect the characteristics of young male participants and should not be generalized to females. To reduce inter-individual variability and improve between-group comparability, the initial posture was standardized. However, this laboratory control may constrain natural sit-to-stand strategies and reduce ecological validity; therefore, the findings reflect biomechanical differences under controlled conditions rather than fully natural movement behavior. Future studies will recruit a larger and sex-balanced sample and perform sex-stratified analyses to eliminate potential confounding effects and improve the generalizability of the findings.

In the present exploratory study, participants were classified according to standardized BMI criteria. However, body composition (e.g., body fat percentage) and muscle strength were not directly measured. We acknowledge that differences in lean mass and strength could partially contribute to the observed biomechanical variations. Future studies will incorporate direct body composition assessment (e.g., bioelectrical impedance or DXA) and strength measurements to better isolate adiposity-related biomechanical effects.

This study relied solely on kinematic and kinetic data for analysis. Moreover, the severe gender imbalance in the study groups and the lack of control over movement speed during the STS task are significant limitations of the present research. The sample in this study was predominantly male, and therefore potential sex-related differences in STS biomechanics were not examined. The findings should be interpreted with caution when generalized. Future research is encouraged to expand the sample size, balance gender distribution, incorporate body composition assessment (e.g., bioelectrical impedance or DXA) and strength measurements, control movement speed, and synergistically incorporate surface electromyography data. This would allow for a more comprehensive characterization of muscle activation patterns during STS, providing deeper insights into the biomechanical strategies of the overweight population.

## 5. Conclusions

The overweight population exhibits different motion characteristics of the hip and knee joints during the STS process compared to the normal-weight population, with more pronounced differences in the hip joint. The hip joint of the overweight population presents a higher demand for balance and stability during STS. Therefore, it is recommended that overweight individuals pay attention to hip joint function maintenance and targeted muscle strength training in daily life and rehabilitation practice. BMI-related movement characteristics should be studied in young adults under controlled experimental conditions, and future studies are needed to verify whether similar patterns exist in older adults.

## Figures and Tables

**Table 1:** Demographic and baseline characteristics of participants (mean ± SD).

	Normal-Weight Group	Overweight Group
Gender (Female/Male)	2/8	1/9
Age (years)	23.6 ± 1.69	24.4 ± 2.46
Height (cm)	172 ± 6.97	176.66 ± 6.85
Body Weight (kg)	62.35 ± 7.91	84.15 ± 8.18
BMI (kg/m^2^)	20.98 ± 1.33	26.92 ± 1.48
MET (min/week)	4629.5 ± 3024.7	3214.4 ± 2139.7

**Table 2:** Temporal parameters between the overweight group and the normal-weight group (mean ± SD).

	Normal-Weight Group	Overweight Group
Total STS time (s)	1.822 ± 0.077	1.739 ± 0.076 *
Pre-seat-off phase duration (s)	0.648 ± 0.054	0.636 ± 0.051
Post-seat-off phase duration (s)	1.174 ± 0.050	1.103 ± 0.040 *

Notes: * indicates statistically significant difference between overweight group and normal-weight group (*p* < 0.05).

**Table 3:** A comparison of joint angle extremes between the overweight group and the normal-weight group (mean ± SD).

Joints	Movement	Peak Value		Peak Value	
		Overweight Group	Normal-Weight Group	Overweight Group	Normal-Weight Group
Hip Joint	Flexion/Extension	62.55 ± 9.82 *	72.09 ± 8.64	−7.09 ± 2.15	−6.15 ± 2.84
	Adduction/Abduction	−5.82 ± 3.19	−3.52 ± 3.39	−17 ± 4.39	−10.54 ± 5.62 *
	Internal Rotation/External Rotation	−3.74 ± 6.02 *	4.31 ± 8.79	−20.79 ± 7.77	−9.8 ± 11.33 *
Knee Joint	Flexion/Extension	87.73 ± 4.82	91.1 ± 4.34	0.18 ± 4.27	−0.63 ± 3.73

Notes: * indicates statistically significant difference between overweight group and normal-weight group (*p* < 0.05).

**Table 4:** Comparison of torque extremes between overweight group and normal-weight group (mean ± SD).

Joints	Movement	Peak Value		Peak Value	
		Overweight Group	Normal-Weight Group	Overweight Group	Normal-Weight Group
Hip Joint	Flexion/Extension	0.11 ± 0.04	0.16 ± 0.06	−0.27 ± 0.08	−0.3 ± 0.14
	Adduction/Abduction	0.03 ± 0.04 *	0 ± 0.03	−0.09 ± 0.04	−0.11 ± 0.04
	Internal Rotation/External Rotation	0.01 ± 0.01	0 ± 0.02	−0.05 ± 0.02	−0.07 ± 0.03
Knee Joint	Flexion/Extension	0.07 ± 0.05	0.08 ± 0.04	−0.34 ± 0.1	−0.42 ± 0.1

Notes: * indicates statistically significant difference between overweight group and normal-weight group (*p* < 0.05).

## Data Availability

The data supporting the findings of this study are available on request from the corresponding author. The data are not publicly available due to privacy and ethical restrictions, as they contain information that could compromise the privacy of research participants.
